# Predicting Poor Outcome of COVID-19 Patients on the Day of Admission with the COVID-19 Score

**DOI:** 10.1155/2021/5585291

**Published:** 2021-05-31

**Authors:** Luke Tseng, Erin Hittesdorf, Mitchell F. Berman, Desmond A. Jordan, Nina Yoh, Katerina Elisman, Katherine A. Eiseman, Yuqi Miao, Shuang Wang, Gebhard Wagener

**Affiliations:** ^1^Columbia University Vagelos College of Physicians & Surgeons, New York, NY, USA; ^2^Department of Anesthesiology, Columbia University Medical Center, New York, NY, USA; ^3^Department of Neurological Surgery, Columbia University Medical Center, New York, NY, USA; ^4^Department of Biostatistics, Mailman School of Public Health, New York, NY, USA

## Abstract

**Background:**

COVID-19 may result in multiorgan failure and death. Early detection of patients at risk may allow triage and more intense monitoring. The aim of this study was to develop a simple, objective admission score, based on laboratory tests, that identifies patients who are likely going to deteriorate.

**Methods:**

This is a retrospective cohort study of all COVID-19 patients admitted to a tertiary academic medical center in New York City during the COVID-19 crisis in spring 2020. The primary combined endpoint included intubation, stage 3 acute kidney injury (AKI), or death. Laboratory tests available on admission in at least 70% of patients (and age) were included for univariate analysis. Tests that were statistically or clinically significant were then included in a multivariate binary logistic regression model using stepwise exclusion. 70% of all patients were used to train the model, and 30% were used as an internal validation cohort. The aim of this study was to develop and validate a model for COVID-19 severity based on biomarkers.

**Results:**

Out of 2545 patients, 833 (32.7%) experienced the primary endpoint. 53 laboratory tests were analyzed, and of these, 47 tests (and age) were significantly different between patients with and without the endpoint. The final multivariate model included age, albumin, creatinine, C-reactive protein, and lactate dehydrogenase. The area under the ROC curve was 0.850 (CI [95%]: 0.813, 0.889), with a sensitivity of 0.800 and specificity of 0.761. The probability of experiencing the primary endpoint can be calculated as *p*=*e*^(−2.4475+0.02492age − 0.6503albumin+0.81926creat+0.00388CRP+0.00143LDH)^/1+*e*^(−2.4475+ 0.02492age − 0.6503albumin+0.81926creat+0.00388CRP+0.00143LDH)^.

**Conclusions:**

Our study demonstrated that poor outcome in COVID-19 patients can be predicted with good sensitivity and specificity using a few laboratory tests. This is useful for identifying patients at risk during admission.

## 1. Introduction

From March to May 2020, New York City experienced a severe crisis of COVID-19 cases that resulted in a surge of patients who required hospital-level care. Hospitals and medical resources were strained to their limits. Although many patients recovered quickly, some progressed to develop severe COVID-19 resulting in significant morbidity and potentially death.

Some features of severe COVID-19 include respiratory failure requiring intubation, severe renal failure, and death. Risk factors for severe disease include advanced age and underlying conditions such as cardiovascular disease and diabetes [1]. Patients admitted to the hospital have a case-fatality rate as high as 24%, and patients admitted to the intensive care unit requiring mechanical ventilation have a case-fatality rate as high as 60% [1, 2].

There is an urgent need for clinical tools that can identify patients at risk for severe COVID-19 during admission. Previous predictive models have used a combination of demographic data, clinical history, and laboratory data to predict risk for severe disease or mortality in patients [3–6]. However, these studies were largely conducted using modest sample sizes predominantly based in China. Recently, there has been interest in using previously validated non-COVID-19 clinical scores, such as the National Early Warning Score (NEWS), in COVID-19 patients [7, 8]. Although many of these models perform well, they lack a large study cohort and are not based solely on objective and quantifiable data, which can lend bias and inaccuracy to the prediction. We excluded less objective or quantifiable data such as comorbidities or findings during physical exam that have previously been used for other scores such as the Veterans Health Administration COVID-19 (VACO) Index for COVID-19 Mortality [9] or the Quick COVID-19 Severity Index (qCSI) [10].

There remains a need for a simple, objective prognostic model that is generalizable to Western populations. Laboratory tests are a promising source of objective data, and there is evidence that inflammatory markers (e.g., C-reactive protein) and markers of cardiac, liver, and renal dysfunction are associated with severe COVID-19 [11, 12]. The aim of this study is to determine which laboratory values on hospital admission can predict poor outcome in COVID-19 patients and to create a predictive COVID-19 score that can help practitioners triage patients on admission to the hospital.

## 2. Materials and Methods

In this retrospective cohort study, we included all patients who were admitted to Columbia University Irving Medical Center during the COVID-19 crisis in New York City from March 10, 2020, to May 24, 2020. COVID-19 positivity was defined as patients who tested positive for SARS-COVID-2 using polymerase chain reaction and who received an admitting diagnosis of COVID-19.

The primary combined endpoint included either intubation, stage 3 acute kidney injury (AKI) defined by Kidney Disease Improving Global Outcomes (KDIGO) criteria [13] (increase in serum creatinine to 3 times the baseline or to ≥4 mg/dL within seven days after admission), or death during hospitalization. We chose these endpoints because we considered the presence of any of these endpoints representative of a serious deterioration of COVID-19 especially during the surge in New York City at that time. We did not include ICU admission because at that time only patients who required mechanical ventilation were admitted to the ICU because of the shortage of ICU beds. Furthermore, due to the lack of ICU beds, a number of mechanically ventilated patients were cared for in improvised ICUs at that time. We also did not include renal failure requiring renal-replacement therapy (RRT), as many patients who under normal circumstances would have required RRT did not receive it due to a lack of RRT machines and/or disposables.

Data were retrieved from electronic medical record systems. We analyzed 99 different laboratory tests and basic demographic information, mainly age and sex. Admission laboratory results were defined as the mean value of results for each patient from one day before to one day after admission.

We first conducted univariate analysis on laboratory tests that were available on admission in ≥70% of patients. Variables that were statistically and clinically significant were included in a multivariate binary logistic regression model. We removed patients who were missing any of these variables, which yielded a final cohort of *n* = 1492 patients. To select the best prediction model for the primary endpoint, 70% of patients (*n* = 1045) were used to train the model. We used a cross-validation (CV) procedure in which 70% of the 1045 training samples (*n* = 732) were used to determine the list of variables to be selected in the model, and the remaining 30% (*n* = 313) were used to examine the model fit. This method was in accordance with type 1b prediction model studies as described by the Transparent Reporting of a multivariable prediction model for Individual Prognosis Or Diagnosis (TRIPOD) Statement (“development of a prediction model using the entire data set, but then using resampling (e.g., bootstrapping or cross-validation) techniques to evaluate the performance and optimism of the developed model”) [14]. We did not include any external validation cohorts.

**Figure 1:**
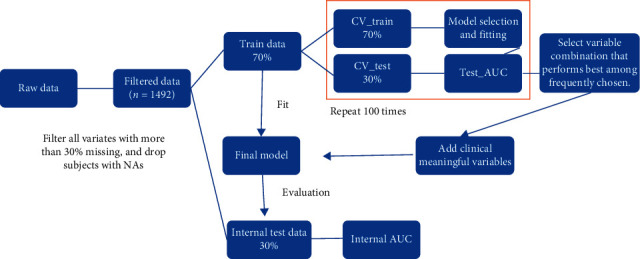
Flowchart depicting how the COVID-19 score was developed.

This CV process was repeated 100 times, and we selected variables that were most frequently selected out of the 100 CVs. This list of variables was considered in the final model and fitted using all *n* = 1492 training samples. The fitted final model was then tested in the internal validation cohort, which consisted of 30% of the cohort (*n* = 447) (Figure 1). Bootstrap samplings were conducted on the internal validation cohort to provide confidence intervals. The final model that was generated for the primary endpoint was also fitted against other specific endpoints (intubation, stage 3 AKI, or death) to estimate the probability of experiencing a specific event. The best cutoff value for the predicted probability was calculated as the value that maximized Youden's J statistic, defined as J = sensitivity + specificity − 1.

We constructed Kaplan–Meier curves to compare event-free survival (in which the event was defined as the primary combined endpoint) between patients with a higher and lower probability than the best cutoff. We created Kaplan–Meier survival curves for patients who were and who were not intubated and for patients with and without stage 3 AKI.

SAS 9.1 (SAS Inc., Cary, North Carolina), PASW 18.0 (SPSS Inc., Chicago, Illinois), R Project for Statistical Computing, and GraphPad Prism 6.0 (San Diego, California) software were used for statistical analysis.

## 3. Results

A total of 2545 patients were admitted to the hospital with COVID-19. 537 patients (21.1%) died, 309 patients (12.1%) were intubated, and 324 patients (12.7%) experienced stage 3 AKI. The primary combined endpoint (intubation, stage 3 AKI, or death) was seen in 833 patients (32.7%). The incidence of specific endpoints can be found in Table 1.

**Figure 2:**
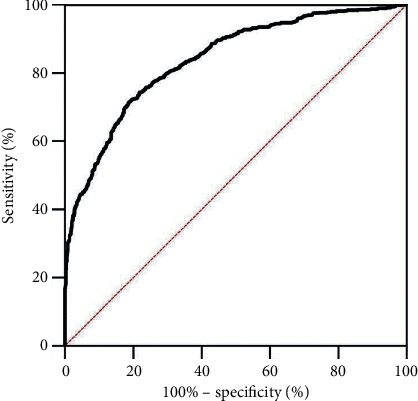
Receiver operating characteristic (ROC) curve of the admission COVID-19 score to predict the primary combined endpoint (either stage 3 acute kidney injury, intubation, or death) in the internal validation cohort (*n* = 447).

Of 99 included laboratory tests (as well as race, sex, and age), 53 were available for ≥70% of patients on admission (Supplementary Table S1). Supplementary Table S2 lists excluded variables. On univariate analysis, 47 variables (and age) were significantly different between patients with and without the combined endpoint. Multivariate analysis yielded a final binary logistic regression model that included age, albumin, creatinine, high-sensitivity C-reactive protein (CRP), and lactate dehydrogenase (LDH). For the primary combined endpoint, the model yielded an area under the receiver operating characteristic curve (AUC) of 0.850 (CI [95%]: 0.813, 0.889) (Figure 2), with a sensitivity of 0.800 and specificity of 0.761 (using a best cutoff of 0.335, as determined by Youden's J statistic). The probability of experiencing any of these outcomes was defined as the COVID-19 score and can be calculated as

(1) $$\begin{array}{c} p=\frac{e^{\left(-2.4475+0.02492\text{age}-0.6503\text{albumin}+0.81926\text{creat}+0.00388\text{CRP}+0.00143\text{LDH}\right)}}{1+e^{\left(-2.4475+0.02492\text{age}-0.6503\text{ albumin}+0.81926\text{creat}+0.00388\text{CRP}+0.00143\text{LDH}\right)}}. \end{array}$$

The model was also fitted for specific endpoints, as shown in Table 2.

**Figure 3:**
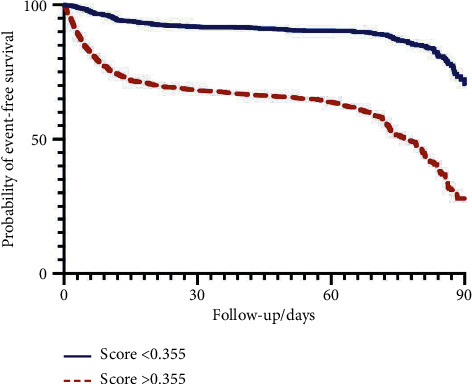
Event-free survival with an admission COVID-19 score above and below 0.355 (determined as the best cutoff by Youden's J statistic). An event is defined as either stage 3 acute kidney injury, intubation, or death.

**Figure 4:**
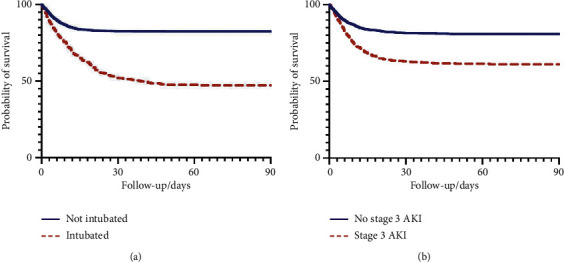
(a) Survival of patients who did/did not require mechanical. (b) Survival of patients with and without stage 3 acute kidney injury.

Patients with a COVID-19 score >0.335 (best cutoff) had a hazard ratio (Mantel–Haenszel) of 3.59 (CI [95%]: 3.136, 4.105) for experiencing the primary combined endpoint. Kaplan–Meier event-free survival curves (in which an event was defined as the primary combined endpoint) are depicted in Figure 3. Kaplan–Meier survival curves were constructed for patients with and without intubation and for patients with and without stage 3 AKI (Figures 4(a) and 4(b)). Patients who required intubation had a hazard ratio (Mantel–Haenszel) of 6.479 (CI [95%]: 5.032, 8.341) for death, and patients with stage 3 AKI had a hazard ratio (Mantel–Haenszel) of 2.837 (CI [95%]: 2.252, 3.574) for death.

The relative contribution of each variable to the model was assessed based on how removal of the variable affected the AUC for predicting the primary endpoint. When creatinine was removed, the AUC decreased from 0.850 to 0.774. When LDH was removed, the AUC decreased from 0.850 to 0.832. When albumin was removed, the AUC decreased from 0.850 to 0.836. When age was removed, the AUC decreased from 0.850 to 0.837. When CRP was removed, the AUC decreased from 0.850 to 0.845.

In previous publications, race, biological sex, and IL-6 level were important predictors of mortality in COVID-19 patients [15, 16]. Although these variables were not selected into our final model, we investigated these variables further by manually adding and removing these variables from the model to determine how they affected the AUC for predicting the primary combined endpoint. Removing race increased the AUC from 0.847 to 0.850. African American race was associated with decreased risk of severe disease (coefficient: −0.344), whereas white race was associated with increased risk (coefficient: 0.450). Removing biological sex minimally increased the AUC from 0.849 to 0.850. Male sex was associated with increased risk for severe disease (coefficient: 0.125). In an earlier iteration of the model, inclusion of IL-6 level (which was only available for 683 patients) did not change the AUC, which was 0.746. Because these variables did not appreciably improve the AUC, they were not included in the final model.

## 4. Discussion

Our study provides clear support that poor outcome of patients with COVID-19 can be predicted on hospital admission with good sensitivity and specificity using a few objective variables. The probability for severe disease (i.e., the COVID-19 score) can be calculated using age, albumin, creatinine, high-sensitivity C-reactive protein (CRP), and lactate dehydrogenase (LDH). The model predicted the primary combined endpoint of intubation, stage 3 AKI, or death with an AUC of 0.850.

The primary advantage of this model is that it uses objective laboratory tests that are commonly available on hospital admission. Although a patient's comorbidities, particularly cardiovascular disease or diabetes, can certainly increase a patient's risk for severe COVID-19, the advantage of using laboratory values (and age) only is that these values are quantitative, easily acquired, and computable data that have a higher degree of objectivity than, for example, comorbidities and which thus lend themselves well to quantitative determination of risk. Previous prognostication models used smaller sample sizes and relied upon a combination of demographic, clinical, radiographic, and laboratory variables that are less easily computable [3–6]. Recently, there has also been interest in applying non-COVID-19 clinical scores, such as the National Early Warning Score (NEWS), to COVID-19 to predict mortality [7, 8]. This approach, however, is also limited by inclusion of data in the score that may be less objective and more difficult to quantify, such as assessment of mental status. Our model provides a higher degree of objectivity by using objective laboratory data that have been tested in a large cohort of over one thousand patients. The use of computable variables only may provide an opportunity to have this score calculated automatically by electronic medical record systems that could flag patients at risk without the need for calculation by healthcare workers.

In our model, creatinine was the variable with the strongest impact on the AUC of our model for predicting the primary endpoint. Although it is unsurprising that creatinine was useful for predicting an endpoint that includes AKI, it is important to highlight two points. First, the creatinine on admission was able to predict not only the development of AKI but specifically progression to severe AKI during the hospital course. Second, creatinine was useful for predicting other endpoints as well, particularly death (removal of creatinine decreased AUC from 0.837 to 0.828).

The association between AKI and death can be seen in our survival curves, which showed that patients with stage 3 AKI had increased mortality. This is consistent with previous studies showing an association between AKI and mortality in COVID-19 patients [17–19]. AKI may be a proxy for severe systemic inflammation and organ dysfunction. Chronic kidney disease (CKD) has also been associated with increased mortality in COVID-19, with increased risk for AKI requiring renal-replacement therapy [20]. The risk for worse outcome may be related to the immunosuppressed state of CKD. Although we are unable to distinguish between AKI and CKD in this study, elevated creatinine on admission is clearly associated with poor outcome.

CRP and albumin, which are acute phase reactants, are associated with severe COVID-19, a highly inflammatory state. CRP is a positively regulated acute phase reactant, whereas albumin is negatively regulated, which explains its negative correlation with severe disease. Higher CRP/albumin ratios have been associated with increased mortality in critically ill patients [21], older adults [22], and septic patients [23]. More recently, high CRP and low albumin have been associated with increased mortality in COVID-19 [16, 24]. Our results are consistent with these findings.

LDH was found to be associated with severe COVID-19. Elevated LDH levels have been associated with increased risk of deterioration and development of critical illness [5, 25]. Elevated LDH levels have also been seen in severe acute respiratory syndrome and Middle East Respiratory Syndrome, which are caused by different strains of coronavirus [26, 27]. A possible explanation for this association is that elevated serum LDH is an indicator of diffuse tissue damage seen in severe disease.

The only demographic variable that was included in the final model was age, which has previously been associated with increased risk for severe COVID-19 [1]. Although other demographic parameters, including biological sex and race, did not significantly improve the model and were ultimately excluded, it is interesting that African American race was associated with lower risk for severe disease compared to white race, which seems inconsistent with previous studies showing increased mortality from COVID-19 in African Americans [15]. One possible explanation for this paradoxical finding is a confounding effect with creatinine. Given that, for the same creatinine, African Americans are estimated to have a higher glomerular filtration rate than white Americans, the negative coefficient for African American race may have acted as a correction mechanism for higher creatinine.

Interestingly, IL-6 and D-dimer levels did not improve the predictive power of our model. Elevated serum IL-6 levels on admission have been associated with severe COVID-19 [16], and IL-6 is thought to underlie the pathogenesis of the cytokine release syndrome responsible for high mortality in COVID-19 [28]. Inclusion of IL-6 did not improve our model, likely because IL-6 levels were available on admission for less than one-third of our cohort, which may have limited the power of our study for detecting the effect of IL-6 on outcome.

D-dimer levels, which reflect hypercoagulability, have previously been associated with poor outcome in COVID-19 when acquired on admission [29]. However, D-dimer level was not statistically significant on univariate analysis, so it was not included in multivariate analysis. Interestingly, D-dimer level was elevated in both patients with and without poor outcome (6.33 *μ*g/mL and 4.95 *μ*g/mL, respectively). Given that an important threshold value for D-dimer level to predict mortality in COVID-19 patients is 2.0 *μ*g/mL [29], one possible explanation for our null finding is that there is no significant difference in additional risk once D-dimer level is above this threshold.

Some limitations of our study must be recognized. Our model predicted the probability of intubation relatively poorly, with an AUC of 0.713, which suggests that standard laboratory tests on admission are only partially predictive of intubation. One possible explanation may be that a substantial number of patients who required intubation from a clinical standpoint were not intubated and subsequently expired, as their families had opted for palliative care. Another possible explanation is that nonlaboratory data, such as oxygen saturation and findings on chest radiography, may be more predictive of intubation [30].

Another limitation is that some laboratory tests, such as D-dimer and IL-6 level, were only available on admission for a minority of patients. This may have limited the power of our study for determining any effect on outcome associated with these variables and may explain why these variables, which have been cited in the literature for their prognostic value, did not add predictive power to our model.

A further limitation of the score is the lack of external validation. We developed the score using a specific cohort in one hospital during the New York City surge, and its applicability needs to be verified using an external cohort in other environments to be widely generalizable. While we chose the endpoints based on our clinical experience during the New York City surge, other endpoints may be useful for practitioners. Specifically, we did not include ICU admission because at that time only mechanically ventilated patients were admitted to the ICU, and some mechanically ventilated patients were cared for in improvised ICUs [31]. We recognize that ICU admission under different circumstances could be a very useful endpoint for practitioners. Any future external validation study could explore additional endpoints such as deterioration of respiratory status (not necessarily requiring mechanical ventilation), ICU admission, or renal failure requiring RRT. This future external validation should include a large cohort with geographic and socioeconomic diversity of medical systems in order to prove its generalizability.

Finally, although our goal was to develop a model based on laboratory tests, it is conceivable that the AUC of our model can be improved further by incorporating patient comorbidities, such as cardiovascular disease or diabetes mellitus. Unfortunately, this data could not be automatically retrieved from our electronic medical record systems, and accessing this information manually for each patient was not feasible.

Our study describes an easily computable model based on biomarkers that can predict poor outcome in patients with COVID-19 on the day of admission. This model may help internists and admitting healthcare providers identify patients at risk and can be computed by electronic medical record systems without any human intervention.

## 5. Conclusions

Our multivariate model can be used to calculate a risk score for severe COVID-19 using only a handful of objective variables on the day of admission. This can serve as an important tool for triaging patients presenting to the hospital with COVID-19. Whether the score is used quantitatively to yield a specific probability or whether it is used in relation to the best cutoff values, we hope the COVID-19 score can aid in the early clinical assessment of COVID-19 patients and improve outcomes.

## Figures and Tables

**Table 1:** Incidence of endpoints (of 2545 admitted patients).

Endpoint	*N*	%
Death	289	11.4
Intubation	124	4.9
Stage 3 AKI	132	5.2
Death/intubation	96	3.8
Death/stage 3 AKI	73	2.9
Intubation/stage 3 AKI	40	1.6
Death/intubation/stage 3 AKI	79	3.1
TOTAL (primary combined endpoint)	**833**	**32.7**

**Table 2:** Prediction results in the internal test set (*n* = 447) using the logistic regression model developed in the training data (*n* = 1492).

	Death intubation AKI stage 3	Death intubation	Death	Intubation	Stage 3 AKI
AUC	0.850 CI [95%]: 0.813, 0.889	0.888 CI [95%]: 0.859, 0.921	0.837 CI [95%]: 0.802, 0.878	0.713 CI [95%]: 0.645, 0.782	0.950 CI [95%]: 0.923, 0.977
Best cutoff (determined by Youden's J statistic)	0.335	0.272	0.258	0.207	0.184
Sensitivity for best cutoff	0.800	0.859	0.794	0.533	0.820
Specificity for best cutoff	0.761	0.775	0.739	0.809	0.930
Sensitivity for cutoff 0.5	0.627	0.646	0.412	0.017	0.689
Specificity for cutoff 0.5	0.872	0.897	0.925	0.979	0.984

*Parameter estimates*					
(Intercept)	−2.4475	−4.7252	−4.0251	1.11234	−4.727
Age (years)	0.02492	0.0475	0.0677	−0.0137	−0.0147
Albumin (g/dL)	−0.6503	−0.6021	−0.9424	−0.7729	−0.0336
Serum creatinine (mg/dl)	0.81926	0.9859	0.076	0.02069	1.46726
High-sensitivity C-reactive protein	0.00388	0.00429	0.00403	0.00238	0.0047
Lactate dehydrogenase (LDH)	0.00143	0.00113	0.00121	0.00083	0.00067

## Data Availability

The datasets used and/or analyzed during the current study are available from the corresponding author on reasonable request.

## Supplementary Materials

Supplementary Materials Table S1: Univariate analysis of laboratory values for patients with and without the combined endpoint (death intubation, stage 3 AKI). Table S2: Excluded variables. Less than 70% of patients had a result on admission for these variables.
