# Supplementary material for: Predicting Poor Outcome of COVID-19 Patients on the Day of Admission with the COVID-19 Score
Source: Crit Care Res Pract. 2021 May 31;2021:5585291. doi: 10.1155/2021/5585291 (PMC8189812; doi:10.1155/2021/5585291)
Supplement: Supplementary Materials — Table S1: Univariate analysis of laboratory values for patients with and without the combined endpoint (death intubation, stage 3 AKI). Table S2: Excluded variables. Less than 70% of patients had a result on admission for these variables. [file 5585291.f1.zip › 5585291.f1/Table S1 Univariate analysis.docx]

**Table S1:** Univariate analysis of laboratory values for patients with and without the combined endpoint (death intubation, stage 3 AKI)

|  | Death, Intubation, Stage 3 AKI | | | No Primary endpoint | | |  |
| --- | --- | --- | --- | --- | --- | --- | --- |
|  | N | Mean | SD | N | Mean | SD | P value |
| Age | 863 | 70.2 | 15.8 | 1682 | 59.7 | 18.9 | <0.0001 |
| Albumin | 814 | 3.31 | 0.54 | 1509 | 3.63 | 0.51 | <0.0001 |
| AlkPhos | 814 | 103.1 | 94.3 | 1509 | 96.1 | 75.4 | 0.053 |
| ALT | 809 | 65.4 | 194.2 | 1509 | 45.4 | 121 | <0.005 |
| anion gap | 846 | 17.9 | 3.8 | 1586 | 15.6 | 2.4 | <0.0001 |
| AST | 813 | 105.9 | 317.1 | 1509 | 57.4 | 152.1 | <0.0001 |
| basophils % | 726 | 0.228 | 0.216 | 1380 | 0.273 | 0.242 | <0.0001 |
| basophils absolute | 725 | 0.0213 | 0.0225 | 1378 | 0.0214 | 0.0224 | 0.94 |
| Bili-dir | 810 | 0.383 | 0.87 | 1491 | 0.265 | 0.648 | <0.0001 |
| Bili-indir | 789 | 0.329 | 0.269 | 1421 | 0.363 | 0.32 | <0.05 |
| Bili-tot | 814 | 0.708 | 1.037 | 1509 | 0.617 | 0.878 | <0.05 |
| BUN | 847 | 43.9 | 31.2 | 1586 | 20.3 | 15.5 | <0.0001 |
| Ca-total | 847 | 8.49 | 0.72 | 1586 | 8.78 | 0.57 | <0.0001 |
| Cl | 848 | 102.6 | 8.7 | 1586 | 100.7 | 5.9 | <0.0001 |
| CO2 | 847 | 21.5 | 4.2 | 1586 | 22.9 | 3.3 | <0.0001 |
| Creat | 847 | 2.95 | 3.02 | 1586 | 1.07 | 0.56 | <0.0001 |
| Creat | 847 | 2.95 | 3.02 | 1586 | 1.07 | 0.56 | <0.0001 |
| CRP-hs | 747 | 170.6 | 93.6 | 1306 | 117.6 | 86.6 | <0.0001 |
| eGFR-AA | 847 | 39.5 | 20.7 | 1585 | 56 | 9.3 | <0.0001 |
| eGFR-non AA | 847 | 36.4 | 20.8 | 1585 | 53.8 | 11.2 | <0.0001 |
| Eosinophils % | 729 | 0.396 | 1.173 | 1403 | 0.686 | 1.447 | <0.0001 |
| Eosinophils absolute | 729 | 0.0326 | 0.0922 | 1403 | 0.052 | 0.1252 | <0.0001 |
| Globulin | 814 | 3.32 | 0.68 | 1509 | 3.19 | 0.58 | <0.0001 |
| Glucose | 847 | 176.4 | 91.3 | 1585 | 143.2 | 68.7 | <0.0001 |
| Hb | 845 | 11.9 | 2.4 | 1661 | 12.5 | 2 | <0.0001 |
| Hct | 845 | 36.5 | 7.1 | 1661 | 37.7 | 5.7 | <0.0001 |
| IMMATURE GRANULOCYTES % | 710 | 1.01 | 0.85 | 1360 | 0.79 | 0.75 | <0.0001 |
| IMMATURE GRANULOCYTES, ABSOLUTE | 708 | 0.112 | 0.15 | 1354 | 0.075 | 0.111 | <0.0001 |
| INR | 756 | 1.37 | 0.97 | 1280 | 1.18 | 0.48 | <0.0001 |
| K | 848 | 4.55 | 0.74 | 1586 | 4.33 | 0.57 | <0.0001 |
| LDH | 724 | 625.3 | 440.5 | 1288 | 418.1 | 265.2 | <0.0001 |
| Lymphocytes % | 811 | 12.7 | 9.3 | 1494 | 17.3 | 9.2 | <0.0001 |
| Lymphocytes absolute | 811 | 1.2 | 2.53 | 1494 | 1.32 | 2.57 | 0.295 |
| MCH | 845 | 29.3 | 2.6 | 1661 | 29 | 2.5 | <0.005 |
| MCHC | 845 | 32.7 | 1.5 | 1661 | 33.1 | 1.3 | <0.0001 |
| MCV | 845 | 89.4 | 7.1 | 1661 | 87.5 | 6.4 | <0.0001 |
| Monocytes % | 807 | 6.32 | 4.11 | 1491 | 7.58 | 4.31 | <0.0001 |
| Monocytes absolute | 807 | 0.55 | 0.427 | 1491 | 0.609 | 1.549 | 0.29 |
| MPV | 820 | 11 | 1.1 | 1634 | 10.7 | 1 | <0.0001 |
| Na | 848 | 140.7 | 7.8 | 1586 | 138.9 | 5.4 | <0.0001 |
| Neutrophils % | 740 | 74.6 | 19.2 | 1405 | 70.1 | 16.9 | <0.0001 |
| Neutrophils absolute | 811 | 7.86 | 4.74 | 1495 | 6.14 | 3.58 | <0.0001 |
| NUCLEATED RBC ABSOLUTE | 845 | 0.0274 | 0.1809 | 1659 | 0.0154 | 0.2386 | 0.199 |
| NUCLEATED RBC AUTO | 845 | 0.234 | 0.947 | 1659 | 0.107 | 1.329 | <0.05 |
| Platelets | 844 | 213 | 97.8 | 1660 | 234.9 | 99.1 | <0.0001 |
| Procalcitonine | 750 | 6.91 | 34.24 | 1324 | 1.01 | 8.18 | <0.0001 |
| Protein-tot | 814 | 6.63 | 0.84 | 1509 | 6.81 | 0.71 | <0.0001 |
| PT | 756 | 16.5 | 7.1 | 1280 | 14.8 | 3.7 | <0.0001 |
| PTT | 744 | 40.1 | 17.4 | 1266 | 35.8 | 12.4 | <0.0001 |
| RBC count | 845 | 4.1 | 0.85 | 1661 | 4.32 | 0.7 | <0.0001 |
| RED CELL DIST WIDTH (RDW) | 844 | 14.8 | 2.1 | 1661 | 14.1 | 1.9 | <0.0001 |
| Troponin hs | 724 | 108.8 | 255.3 | 1283 | 29.8 | 88.4 | <0.0001 |
| WBC | 845 | 9.93 | 6.38 | 1661 | 8.23 | 4.86 | <0.0001 |
