# Supplementary material for: Predicting Poor Outcome of COVID-19 Patients on the Day of Admission with the COVID-19 Score
Source: Crit Care Res Pract. 2021 May 31;2021:5585291. doi: 10.1155/2021/5585291 (PMC8189812; doi:10.1155/2021/5585291)
Supplement: Supplementary Materials — Table S1: Univariate analysis of laboratory values for patients with and without the combined endpoint (death intubation, stage 3 AKI). Table S2: Excluded variables. Less than 70% of patients had a result on admission for these variables. [file 5585291.f1.zip › 5585291.f1/Table S2 Excluded variables. .docx]

**Table S2:** Excluded variables. Less than 70% of patients had a result on admission for these variables.

| **Variable** | **n** |
| --- | --- |
| Erythrocyte Sedimentation Rate | 1804 |
| D-dimer | 1742 |
| Lactate | 1669 |
| Base Excess | 1493 |
| IL-6 | 1430 |
| pH-venous | 1295 |
| pCO2-venous | 1295 |
| pO2-venous | 1295 |
| SvO2 | 1294 |
| HCO3-venous | 1290 |
| temp | 1010 |
| pH-venous (temp corr) | 861 |
| pCO2-venous (temp-corr) | 861 |
| pO2-venous (temp corr) | 861 |
| B-Natriuretic Peptie | 853 |
| BUN/Creat ratio | 725 |
| Ca-ionized | 657 |
| SEGMENTED NEUTROPHILS %, MANUAL | 509 |
| TOT CELL COUNT/DIFF | 509 |
| pH | 464 |
| HCO3 | 464 |
| pCO2 | 464 |
| SaO2 | 456 |
| pO2 | 456 |
| Fibrinogen | 421 |
| Bands % | 316 |
| pH (temp corr) | 314 |
| pCO2 (temp-corr) | 314 |
| pO2 (temp corr) | 314 |
| ATYPICAL LYMPHOCYTES %, MANUAL | 308 |
| megakaryocytes % | 150 |
| CHOLESTEROL/HDL RATIO | 116 |
| HDL CHOLESTEROL | 116 |
| CHOLESTEROL, TOTAL | 116 |
| TRIGLYCERIDES, SERUM | 116 |
| LDL CHOLESTEROL/CALC | 111 |
| nucleated RBC | 42 |
| HYPERSEG NEUTROPHILS | 12 |
| PROMYELOCYTES %, MANUAL | 11 |
| BLASTS %, MANUAL | 5 |
